# Highly stable, extremely high-temperature, nonvolatile memory based on resistance switching in polycrystalline Pt nanogaps

**DOI:** 10.1038/srep34961

**Published:** 2016-10-11

**Authors:** Hiroshi Suga, Hiroya Suzuki, Yuma Shinomura, Shota Kashiwabara, Kazuhito Tsukagoshi, Tetsuo Shimizu, Yasuhisa Naitoh

**Affiliations:** 1 Department of Technology of Chiba Institute of Technology, 2-17-1 Tsudanuma, Narashino, Chiba 275-0016, Japan; 2 International Center for Materials Nanoarchitectonics (WPI-MANA), National Institute for Materials Science (NIMS), Namiki 1-1, Tsukuba, Ibaraki 305-0044, Japan; 3 Nanomaterials Research Institute, Department of Materials and Chemistry, National Institute of Advanced Industrial Science and Technology (AIST), Higashi 1-1-1 Tsukuba, Ibaraki 305-8562, Japan; 4 Nanoelectronics Research Institute, Department of Electronics and Manufacturing, National Institute of Advanced Industrial Science and Technology (AIST), Higashi 1-1-1 Tsukuba, Ibaraki 305-8562, Japan

## Abstract

Highly stable, nonvolatile, high-temperature memory based on resistance switching was realized using a polycrystalline platinum (Pt) nanogap. The operating temperature of the memory can be drastically increased by the presence of a sharp-edged Pt crystal facet in the nanogap. A short distance between the facet edges maintains the nanogap shape at high temperature, and the sharp shape of the nanogap densifies the electric field to maintain a stable current flow due to field migration. Even at 873 K, which is a significantly higher temperature than feasible for conventional semiconductor memory, the nonvolatility of the proposed memory allows stable ON and OFF currents, with fluctuations of less than or equal to 10%, to be maintained for longer than eight hours. An advantage of this nanogap scheme for high-temperature memory is its secure operation achieved through the assembly and disassembly of a Pt needle in a high electric field.

High-temperature electronics, which can be operated at extremely high temperatures, are wanted to reduce the energy required for cooling and for the direct sensing of extremely high-temperature information. In addition to the energy savings, sufficient endurance for high-temperature operation is enormously beneficial for suppressing thermal runaway due to thermal activation loss in the electric circuit. Furthermore, high-temperature memory can preserve valuable high-density data even when high-temperature heating damage affects a memory device^1^. We cannot currently expect to obtain direct digital information in cases of fire accidents because there are few technologies that can preserve digital data at high temperatures. Such high-temperature memories for devices such as flight data recorders for aircraft, drive recorders for vehicles, and robot systems for emergency searches would contribute to improvements in reliability engineering. Furthermore, high-temperature memories in applications such as planetary probes would be able to store important information recorded under high-temperature conditions, which would open the doors to a new scientific and technological world. However, such desires are in opposition to the current direction of development of state-of-the-art memory, which demands high density and rapid operation achieved through miniaturization of the semiconducting channel and thinner tunneling barriers and is designed for operation at conventional temperatures, without any additional temperature stability margin. Typical silicon-based memory is limited to operating temperatures below 473 K because the semiconductors cannot retain their functionality at high temperatures once the number of thermally induced carriers increases to the level of the doping concentration^2^. The number of thermally induced carriers depends on the semiconductor band gap^3^.

Among the candidates for high-temperature memory, for non-volatile memory elements, flash memory has been developed^4^. The maximum temperature for the stable operation of flash memory has been reported to be 443 K^5^. For operation at higher temperatures, alternative operation schemes are required in place of conventional field-effect operation for switching between two discrete current states (ON and OFF). Electric memory also has difficulty operating at high temperatures because such memory requires improvements in the endurance of the electric storage cells in addition to the switching transistors. An alternative scheme involves two electrodes facing together, separated by a nano- or decananoscale gap to control electron tunneling. Such nanogaps can be used to investigate the electrical properties of nanosized materials, such as molecules and nanoparticles^6^,^7^,^8^, and have been found to exhibit characteristic phenomena that are of potential use in nanophotonics and nanoelectronics^9^,^10^,^11^.

Two electrodes facing together and separated by a nano- or decananoscale gap produce ON and OFF states as follows^12^,^13^,^14^. When a bias voltage is applied to the two electrodes through the nanogap, a small portion of one electrode element migrates into the nanogap space in the electric field, forming an atomic-scale needle to generate a current flow through the nanogap^15^. Although the conduction is based on a tunneling current in this case, a clear ON-state current can be generated, in contrast to the current-suppressive state, which serves as an OFF state. To turn off the conductive current, a pulsed voltage inducing a high-density current stimulates the disassembly of the emissive needle element in the nanogap. This switching between the conductive and suppressive states is referred to as nanogap resistance switching (NGS) and allows the structure to act as a switching and/or memory element. Under a low bias voltage, which is typically an order of magnitude smaller than the voltage for needle formation/disassembly, only a low-density tunneling current is generated for reading whether the current state is conductive or suppressive. The low-density tunneling current maintains the conductive/suppressive state as long as the local nanostructure of the nanogap surfaces of the electrodes maintains its shape.

The switching characteristic strongly relies on the electrode material, the nanogap size, and the crystallinity^12^,^13^,^16^,^17^. The typical switching speed and retention time of NGS at room temperature (293–297 K) are shorter than 1 μs and longer than several years, respectively^12^,^18^. In general, NGS at high temperatures requires a material with a high melting temperature, although there is a trade-off with the switching voltage^16^. To maintain stable ON/OFF operation, high-temperature stability requires a sharp edge of the high-melting-temperature material and a very small gap space. As a possible material that can satisfy these requirements, platinum (Pt) crystals could serve as suitable electrodes for NGS.

Various techniques employing mechanically controlled break junctions (MCBJs)^19^,^20^,^21^, electron-beam lithography^22^, chemical etching^23^,^24^, and other methods have been developed to fabricate nanogap electrodes^25^,^26^,^27^,^28^. In this study, we used a Pt nanogap, which has a melting temperature higher than that of the Au nanogaps that are commonly used for NGS^29^. Further treatment for the formation of the polycrystalline Pt allowed us to shape the nanogap, intensifying the electric field for the reproducible formation of elements in the nanogap, even at high temperatures. The polycrystalline Pt formed multiple facets at the grain boundaries in the electrodes, resulting in a drastic elevation of the NGS operating temperature up to 873 K. Clear ON/OFF operation was maintained for eight hours, even at 873 K, with very small fluctuations in resistance. The activation property of the device, analyzed in terms of the Arrhenius behavior, is discussed to elucidate the element migration at the Pt facet edge for NGS.

Nonvolatile nanogap memory was fabricated on 300-nm-thick SiO_2_ that was thermally grown on a Si wafer. Multiple Pt nanowires of 500 nm in width and 5 μm in length were simultaneously patterned via the lift-off technique using high-resolution electron beam lithography. A 5-nm-thick Cr film was deposited via vacuum evaporation, followed by a 30-nm-thick Pt film (Fig. 1(a)). Then, a forming current was applied using a probing station equipped with a source meter (Keithley 2636) to open the gap in the nanowire (Fig. 1(b) and Supplementary Information Fig. S1). For nanogap opening in a Pt nanowire, a controlled atmosphere is critical to reduce gap-size fluctuations. The application of oxygen gas (at a typical gas pressure of 1 × 10^4^ Pa) or hydrogen (at a gas pressure of 1 × 10^4 ^Pa) drastically changes the shape of the Pt nanowire, causing it to become polycrystalline with several crystal boundaries. In this study, hydrogen diluted with Ar (H_2_ content ~4%) was introduced into the closed chamber. This atmosphere enables the reproducible formation of multiple-grain, polycrystalline Pt in nanowires^30^.

As the formation current was gradually increased, a large current heated the continuous nanowire to facilitate element migration in the wire (Fig. 1(b)-i). Once an overloading current caused current saturation, the applied current was shut off to allow the wire to cool before it was blown away, resulting in the growth of multiple crystal grains connected by unexpected necks^31^,^32^,^33^,^34^,^35^. Then, a second applied current would become denser in the necked sections and facilitate electro-migration there, leading to more crystallization along the migrating sections (Fig. 1(b)-ii). After multiple crystal grains had grown in the nanowire, the Pt crystal grains merged into multiple large crystals connected by multiple facets at the grain boundaries in series. Because the further application of a formation current selectively blew away the weakly connected facets, a nanoscale gap faced with two sharp facet edges appeared between the large grains ((Fig. 1(b)-iii,(c))^36^,^37^. Thus, the formed nanogaps were always located at facet edges of the crystal boundaries. Such a facet-edge nanogap has the advantage of suppressing the facile migration of Pt atoms during NGS operation. To measure the ON/OFF ratio during NGS memory operation (Fig. 2(a)), a bias voltage pulse was applied by means of computational routines under vacuum (<10^−4^ Pa) (Fig. 2(b)). For resistance switching applications, our previous study demonstrated a possible means of sealing a device using an inactive gas such as N_2_ or Ar gas and integrating such devices using a vertical nanogap structure^38^,^39^. To obtain the ON state, the bias voltage was gradually increased in 20-mV increments to more than 6 V, followed by a rapid voltage drop to 0 V (Pulse A in Fig. 2(b)). During the increase in the Pulse A voltage, the system compliance of the source meter prevented the current from exceeding 1 μA to prevent damage around the nanogap (Pulse A in Fig. 2(c)). Then, a high electric field with a small current was applied to the nanogap, facilitating Pt-atom migration on the electrode surfaces and the accumulation of Pt atoms in the area with the highest field. The assembled Pt atoms formed an emissive needle in the high-electric-field region (Fig. 2(a)). This emissive needle generated a current flow in the nanogap, and the apparent resistance calculated from the bias voltage and the net current was drastically lowered, by several orders of magnitude, from that observed in the initial ON state. Subsequently, to suppress the electron flow in the nanogap, the bias voltage was again gradually increased in 20-mV increments to more than 6 V, followed by a rapid voltage drop to 0 V (Pulse B in Fig. 2(b,c)). During Pulse B, a large current, typically of 0.5 mA, flowed through the nanogap. This current was three orders of magnitude larger than the compliance current of 1 μA observed during Pulse A. The Pt atoms in the needle migrated away, causing the disassembly of the emissive needle and resulting in the suppression of the current flow in the nanogap. The system compliance of the source meter is the key to the accumulation of Pt atoms for needle formation and disassembly in repeatable NGS memory operation. Further details of the temperature dependence are investigated later in the manuscript. During memory operation, the state with a high generated current is regarded as the low-resistance state (LRS), whereas the state with a suppressed current is the high-resistance state (HRS). To read whether the device is in the LRS or the HRS without destroying the electric state, a low voltage of 0.6 V was applied to limit the low-density tunneling current (Fig. 2(b)). This two-state switching could be repeated through cyclic repetition of the voltage sequence (Fig. 2(d)), and each resistance state, i.e., the HRS or the LRS, could be retained for more than eight hours (Fig. 2(e)).

The temperature dependence of the NGS memory was characterized by applying a controlled current to a ceramic heater located beneath the specimen to produce various temperatures between 303 and 873 K (Fig. 3). Clear NGS was observed in the polycrystalline Pt nanogap between 308 K and 873 K (Figs 2(d) and 3(a); further details of the temperature dependence are provided in Supplementary Information Section 2). The temperature dependences of the HRS and the LRS, averaged over 100 cycles (Fig. 3(a)), are plotted in Fig. 3(b). As a general trend, the HRS and LRS maintained nearly constant OFF and ON currents, respectively, over the entire range of temperature variation, resulting in a constant ON/OFF resistance ratio (Fig. 3(e)). By contrast, a reference nanogap fabricated from Au under vacuum conditions showed an unstable temperature dependence (Fig. 3(c,d)). Although the ON/OFF resistance ratio could be maintained at temperatures up to 400 K, the ON/OFF ratio of the Au nanogap began to decrease during cyclic measurements at 573 K (Fig. 3(c)) because the nanogap was easily deformed during cyclic switching. Eventually, at 600 K, the HRS and LRS of the Au nanogap become nearly indistinguishable. The difference in the temperature dependences between polycrystalline Pt and Au, as shown in Fig. 3(e), is pronounced, suggesting that more reproducible operation at higher temperatures can be expected of polycrystalline Pt NGS memory than of Au NGS memory. The Au nanogap shows less stability than the Pt nanogap. This is because Au has a higher migration rate than Pt^29^. The switching effect strongly depends on the geometry, crystal facets, and size of the nanogap in the Pt nanowire. Therefore, it would be valuable to provide additional statistical information on these characteristics. The gap distances were adjusted at each applied voltage during switching operation. However, at this stage, the crystal size and orientation were randomly formed; consequently, the geometry and crystal facets in the fabricated electrodes could not be controlled. We are presently investigating this issue, and the results will be reported in a future paper. Therefore, as the main finding of the present study, we would like to focus on the resistance switching exhibited by nanogaps in polycrystalline Pt nanowires at 873 K. Figure SI6 illustrates the reproducibility of the individual samples. Although the histograms show some fluctuations, similar switching at 873 K was confirmed for more than 10 samples, which constituted the majority of all fabricated samples; therefore, this resistance switching is thought to be a statistically significant effect.

Here, the transition in polycrystalline Pt NGS is analyzed at various temperatures (Fig. 4). A delayed transition from the HRS to the LRS occurs after the rapid application of a bias voltage of 3 V to the nanogap. This voltage generates a slower transition in comparison with the optimal transition induced by the critical voltage of 4 V, causing the shift in time. The transition at a 4-V bias voltage is much shorter than milliseconds, which is the typical detection limitation. As plotted in Fig. 4(a), the transition delay strongly depends on the temperature. The transition delay time (*t*_c_) from the HRS to the LRS is extended at higher temperature, although the thermal assistance might be intuitively expected to facilitate the formation of an emissive needle in the nanogap via atomic migration. The observed temperature dependence indicates that the thermal effect suppresses the atomic accumulation for needle formation. The plot of *t*_c_ (Fig. 4(b)) extracted from Fig. 4(a) indicates an exponential extension of *t*_c_ with temperature. Based on an assumption of Arrhenius-type thermal activation^40^, this exponential trend can be fitted to


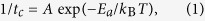


where *A* is a constant, *E*_a_ is the activation energy, and *k*_B_ is the Boltzmann constant. The above equation was successfully fitted to the temperature-dependent *1*/*t*_*c*_ data (inset in Fig. 4(b)). The extracted activation energy *E*_a_ was −10.8 meV. Thus, in terms of the transition from the HRS to the LRS, our operation scheme achieves a greater improvement in transition stability than in the ability to operate at higher temperatures. In addition to the transition from the HRS to the LRS, observation of the transition from the LRS to the HRS was also attempted. However, the value of *t*_c_ was typically shorter than the experimental resolution of our system throughout the entire temperature range between 303 K and 873 K. The model for the resistance transition from the HRS to the LRS is based on an equilibrium phenomenon, which involves a balance between *needle growth and needle destructio*n, since the transition initially fluctuates and then becomes constant (Fig. 4a). The “negative” activation energy is the result of assuming an Arrhenius relationship based on the theory of equilibrium phenomena. *The extracted value of −10.8 meV is the difference between the activation energy for needle growth and that for needle destruction.* This result suggests that the activation energy for needle destruction is larger than that for needle growth. The *E*_*a*_ *value of −10.8 meV* is far lower than the thermal energy at room temperature (25 meV), which indicates that the balance between Pt needle growth and destruction does not drastically change with temperature. This is why resistance switching in a Pt nanogap still occurs at high temperatures.

Here, we consider NGS stability at high temperatures. A structural change in the electrode occurs only on the anode side, as confirmed by direct observations^41^; a small proportion of the Pt atoms accumulate in the region of the highest electric field in the nanogap and form a needle tip when a bias voltage is applied. Once the needle tip is formed, the electric field is further enhanced, causing the tip to grow, as long as no thermal effect stimulates the atoms to disassemble. The field-assisted growth of the tip can be understood as a “field migration” phenomenon^42^,^43^. This field migration occurs in a localized area on the metal surface of the anode. This is why only Pt atoms on the surface undergo field migration and form a needle. Such needle growth as a result of field migration has been confirmed in a previous study using scanning tunneling microscopy (STM)^43^. The growth rate of the needle structures was also clarified^44^. The needle tip behaves as a current-emissive tip, similar to that used in scanning electron microscopy. Consequently, current can densely flow through the nanogap. In contrast to the mechanism of needle formation in the electric field, however, this high-density current flow itself exerts a negative effect on needle formation. The very high-density electrons push the needle back toward the anode side, resulting in the migration of the atoms back onto the anode. This effect is known as “electro-migration” and also as “wind force”^45^,^46^. Furthermore, the high-density current causes Joule heating in/near the nanogap, enhancing the migration of the Pt atoms in the tip. Once the Pt atoms begin to migrate from the tip, the needle disappears. Thus, for the needle formation during the transition from the HRS to the LRS, the switching characteristics in the nanogap depend on the electric field rather than the actual current flow. By contrast, for the needle disassembly during the transition from the LRS to the HRS, a simple high-density current flow removes the atoms from the needle. The removal force exerted by the electro-migration force can be much stronger than the field-migration force in our system under our operating conditions, leading to temperature-insensitive rapid switching for the transition from the LRS to the HRS. According to STM observations using a gold tip, the size of the needle that forms for current emission is typically on the scale of a small cluster of atoms, as reported in ref. 43, and is much smaller than the scale of the gap geometry. Similar to the case for gold, platinum can also form an atomic-cluster-scale needle on the anode-side surface for current emission. Because the scale of the needle is much smaller than that of the gap geometry, the driving force for needle removal is solely dominated by electro-migration.

Here, we consider two nanogaps with different facet edge shapes for comparison (Fig. 5). Although these two nanogaps were prepared using the same fabrication and formation schemes, uncontrollable fluctuations resulted in different shapes of the facet edges in the nanogaps. The nanogap in Fig. 5(b) has a relatively sharp edge, whereas the nanogap in Fig. 5(c) consists of two wide, flat parallel plates. Below 600 K, both nanogaps exhibit reproducible NGS (Fig. 5(a)). At high temperatures, however, the flat-edged nanogap loses its distinct ON/OFF ratio. This degradation indicates that electro-migration is the main driving force in the flat-edged nanogap in the high-temperature regime.

According to a basic electric-field analysis simplified for a two-dimensional conductor, the electric field of the sharp edge is approximately twice as high as that of the flat edge. Under the assumption of a simple tunneling effect without heating behavior, we can extract the structural parameters of our nanogap electrodes from their observed characteristics in non-destructive current switching events, based on the basic tunneling equation, in which the tunneling current is expressed as


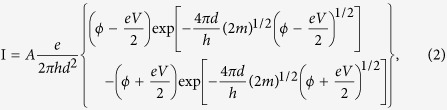


where 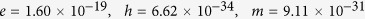, and V, *d, A*, and *ϕ* denote the applied voltage, the gap width, the tunneling emission area, and the barrier height, respectively^47^,^48^,^49^. In Table 1, the fitted parameters of the two nanogaps are listed. Only the tunneling emission area significantly differs. These findings indicate that in a sharp-edged nanogap, the electric field is more highly focused, better maintaining the needle tip against the wind force.

Using the sharp-edged Pt nanogap discussed above, long-term retention measurements were performed (Figs 2(e) and 6). Both the HRS resistance and the LRS resistance measured at 1.0 × 10^−3^ Pa were stable. These resistances did not change over a period of 8 h at 303 K (Fig. 2(e)). The observed resistance fluctuations in the LRS were less than or equal to 10%. At 873 K (Fig. 6), the LRS resistance again did not change over a period of 8 h. Because of the long-term tolerance limitations of our instrument at 873 K, the stability of the NGS memory at 873 K may be much longer than our actual observation.

In summary, we succeeded in the first realization of extremely high-temperature memory based on a polycrystalline Pt nanogap. During operation, this memory can exhibit clear ON/OFF ratio even at 873 K. Memory retention over at least 8 h at extremely high temperatures is observed. To our knowledge, this is the highest operating temperature achieved to date for electric memory. As such, the reported process paves the way for the future development of non-Si-based, high-temperature electronic devices. The temperature dependence of the transition time can be modeled using the Arrhenius equation, which suggests that nanogap memory operation is based on the equilibrium control of Pt-atom accumulation in a high electric field and disassembly driven by Pt-atom migration. Because of this operating mechanism, NGS memory offers the advantage of secure operation for information storage at high temperatures compared with other memories.

## Additional Information

**How to cite this article**: Suga, H. *et al*. Highly stable, extremely high-temperature, nonvolatile memory based on resistance switching in polycrystalline Pt nanogaps. *Sci. Rep.* **6**, 34961; doi: 10.1038/srep34961 (2016).

## Supplementary Material

Supplementary Information

## Figures and Tables

**Figure 1:**
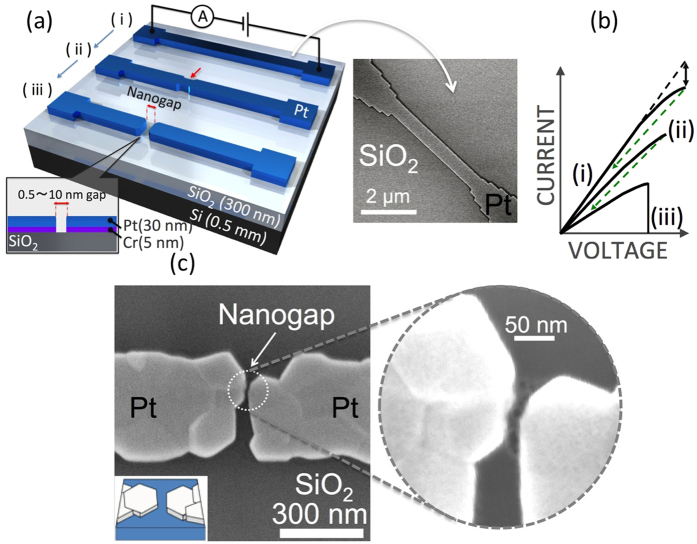
(**a**) Schematic and SEM images of a Pt nanowire fabricated on a SiO_2_/Si substrate. After the formation of multiple nanowires, the nanogaps were opened one by one. (**b**) Nanogap formation sequence. (**c**) SEM image of a Pt polycrystalline nanowire with nanogaps. Excess current in the Ar/H_2_ (4% hydrogen) ambient gas caused the formation of polycrystalline Pt in the nanowire and then opened a nanogap at a facet edge of the polycrystalline Pt. Typically, three-step current application will reproducibly open nanoscale gaps.

**Figure 2:**
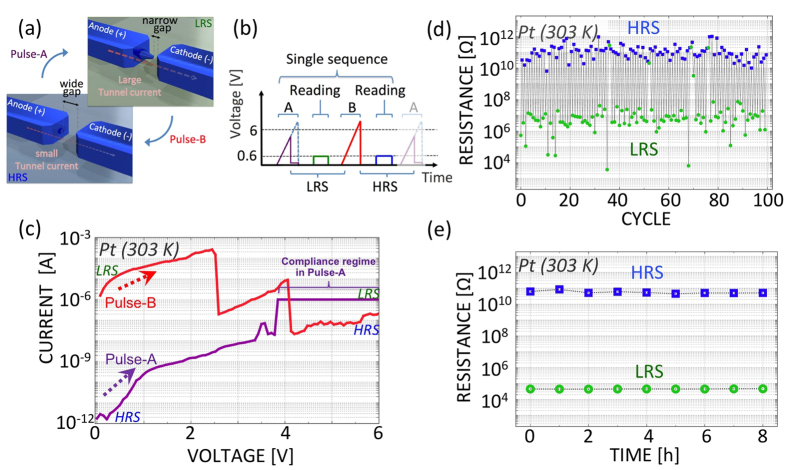
(**a**) Schematic diagram of the switching of a nanogap between the high-resistance state (HRS) and the low-resistance sate (LRS). During the transition from the HRS to the LRS, a current-emissive needle tip is formed in the electric field. During the transition from the LRS to the HRS, this needle tip is disassembled, and the element migrates back onto the electrode under the high-density current flow in the nanogap. To eliminate accidental large currents, which could cause a fatal deformation of the nanogap, the system’s current compliance of 1 μA suppressed excess current during the cyclic repetition of this switching phenomenon in this study. (**b**) Voltage operation sequence for the setting of the HRS and LRS for the reading of the nanogap resistance at desired intervals. (**c**) Typical current transitions from the LRS to the HRS and from the HRS to the LRS at 303 K. (**d**) Typical NGS between the HRS and LRS, measured 100 times. (**e**) Long-term stability of the HRS and LRS at 303 K. Because of the stable structure serving as the foundation of the switching scheme and the non-destructive measurement enabled by the 0.6-V bias voltage, stable and distinct resistance values were maintained in both states, with fluctuations of 10% or less.

**Figure 3:**
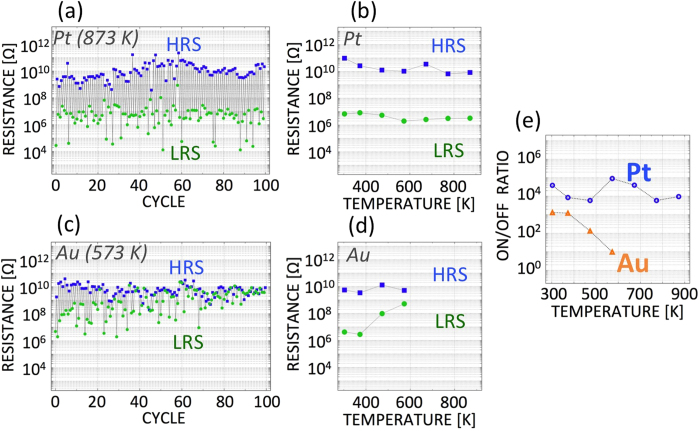
(**a**) Resistance switching in polycrystalline Pt nanogaps at the highest possible temperature of 873 K. (**b**) Temperature dependence of Pt NGS. (**c**) Resistance switching in Au nanogaps at the highest possible temperature of 573 K. (**d**) Temperature dependence of Au NGS. (**e**) Temperature dependences of the ON/OFF ratios for polycrystalline Pt nanogaps and Au nanogaps.

**Figure 4:**
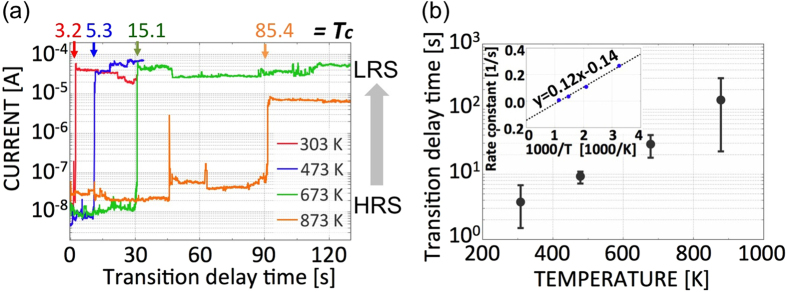
(**a**) Temperature-dependent transition from the HRS to the LRS detected under a continuous bias voltage of 3 V. The transition delay after the application of the bias voltage depends on the temperature. (**b**) Temperature dependence of the transition delay time and an activation energy analysis based on Arrhenius activation. Inset: Arrhenius plot of 1/*t*_*c*_ as a function of the inverse temperature. In this analysis, it is assumed that the formation and migration of the needle tip element are thermally assisted at high temperatures.

**Figure 5:**
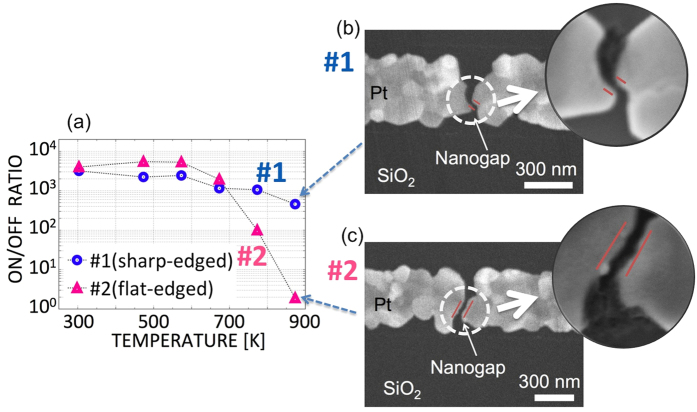
(**a**) Comparison of the ON/OFF ratios at high temperature between a sharp-edged gap shape and a flat-edged gap shape in the polycrystalline Pt nanogap. (**b**) SEM image of the sharp-edged gap. (**c**) SEM image of the flat-edged gap. The sharp edge causes the electric field to increase to up to 1.8 times higher than that in the case of the flat edge.

**Figure 6:**
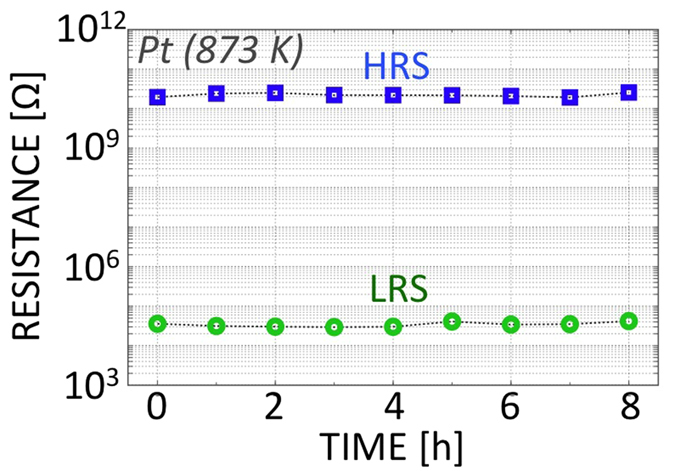
Stability of the HRS and LRS of the sharp-edged Pt nanogap at 873 K, which is the limiting temperature for our equipment.

**Table 1:** Structural sizes and work functions extracted using the basic tunneling equation for the two nanogaps, one with a sharp edge (#1) and one with flat edge (#2), depicted in Fig. 5.

	Resistance *R* [MΩ]	Gap size *d* [nm]	Emission area *A* [nm^2^]	Work function *Φ* [eV]
sample 1 (sharp edge)	16.9	1.36	7.0	0.38
sample 2 (flat edge)	17.9	1.32	12	0.42
