# Supplementary material for: Highly stable, extremely high-temperature, nonvolatile memory based on resistance switching in polycrystalline Pt nanogaps
Source: Sci Rep. 2016 Oct 11;6:34961. doi: 10.1038/srep34961 (PMC5057135; doi:10.1038/srep34961)
Supplement: Supplementary Information [file srep34961-s1.pdf]

Supplementary information

Highly stable, extremely high-temperature, nonvolatile memory using resistance-switching in polycrystalline Pt-nanogaps

Hiroshi Suga, Hiroya Suzuki, Yuma Shinomura, Shota Kashiwabara,
Kazuhito Tsukagoshi, Tetsuo Shimizu, and Yasuhisa Naitoh

Table of Contents

1. Experimental setup for the measurements 1
2. Details of the temperature dependence of the NGS in a polycrystalline Pt nanogap 2
3. Retention characteristics at high temperatures 3
4. Material and nanogap shape dependence of switching reproducibility 4
5. Temperature dependence of resistance 5
6. Device-to-device, cycle-to-cycle uniformity 6

1. Experimental setup for the measurements

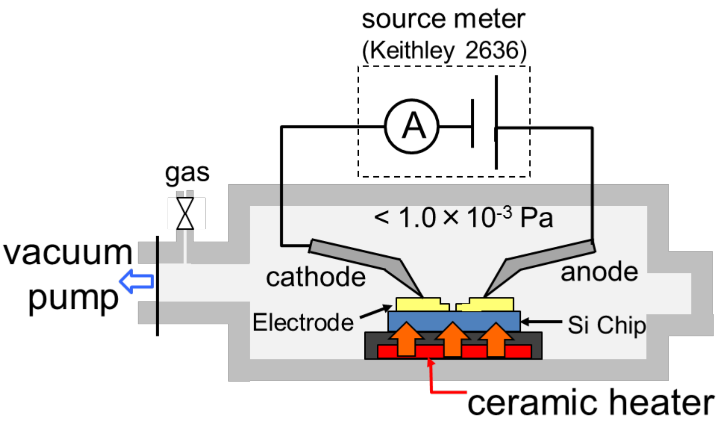

Fig. SI1 Experimental setup for the *I-V* measurements.

The current-voltage (*I-V*) measurements were performed in a closed chamber with a controlled atmosphere. During the cyclic switching measurements presented in Figs. 2(d), 3(a)(c), and SI2 (a)-(g), a compliance current was enforced for Pulse A (Fig. 2(b)) to prevent the fatal destruction of the nanowire and nanogap by an overloading current.

2. Details of the temperature dependence of the NGS in a polycrystalline Pt nanogap

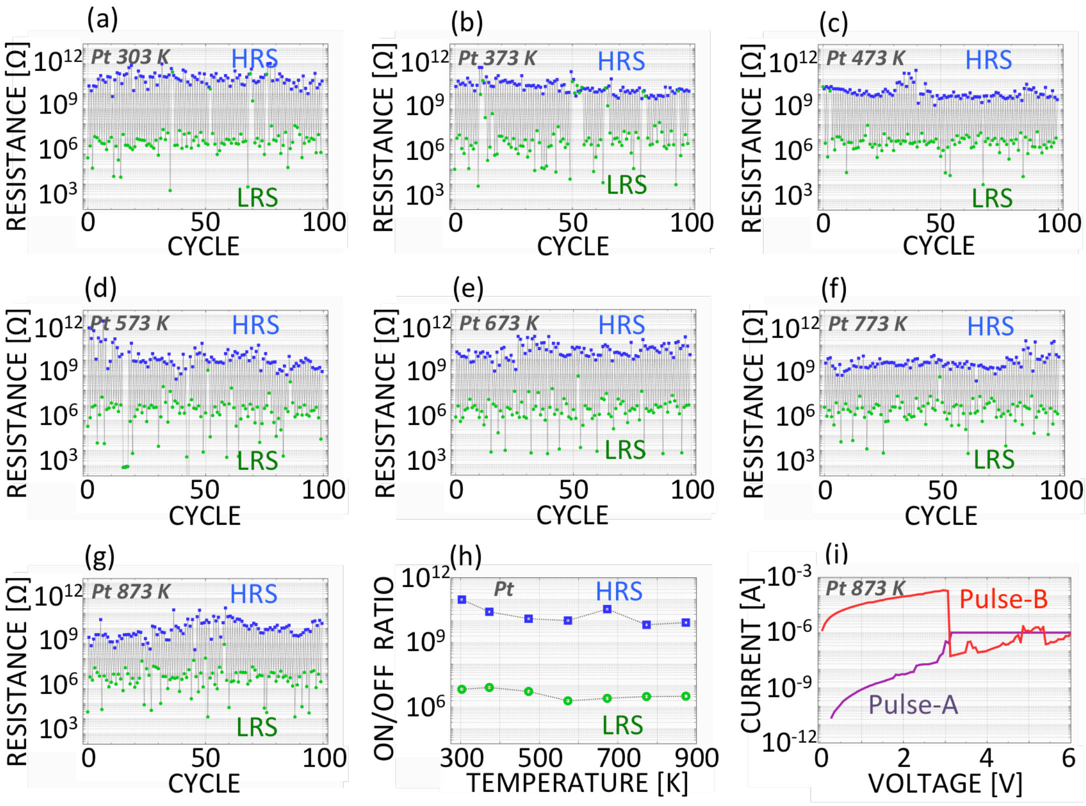

Fig. SI2 (a-g) Cyclic switching observed at (a) 303 K, (b) 373 K, (c) 473 K, (d) 573 K, (e) 673 K, (f) 773 K, and (g) 873 K in a polycrystalline Pt nanogap with a sharp edge shape. (h) Temperature dependences of the resistance values of the HRS and LRS. (i) Transitions from the LRS to the HRS and from the HRS to the LRS observed in I-V measurements at 873 K.

The LRS and HRS in a sharp-edged polycrystalline Pt nanogap were cyclically measured at various temperatures. As an overall trend, no evident change was observed in the basic properties of the switching behavior at different temperatures, as shown in Fig. SI2 (a)-(g). The temperature dependences of the HRS and LRS resistance values

are plotted in Fig. SI2 (h), showing a steady ON/OFF ratio of greater than 10^3. These plots confirm the stability of the switching effect over a wide temperature range. The high-temperature transitions from the LRS to the HRS and from the HRS to the LRS observed in I-V measurements at 873 K (Fig. SI2) are very similar to the low-temperature behaviors observed at 303 K (Fig. 2(c)).

3. Retention characteristics at high temperatures

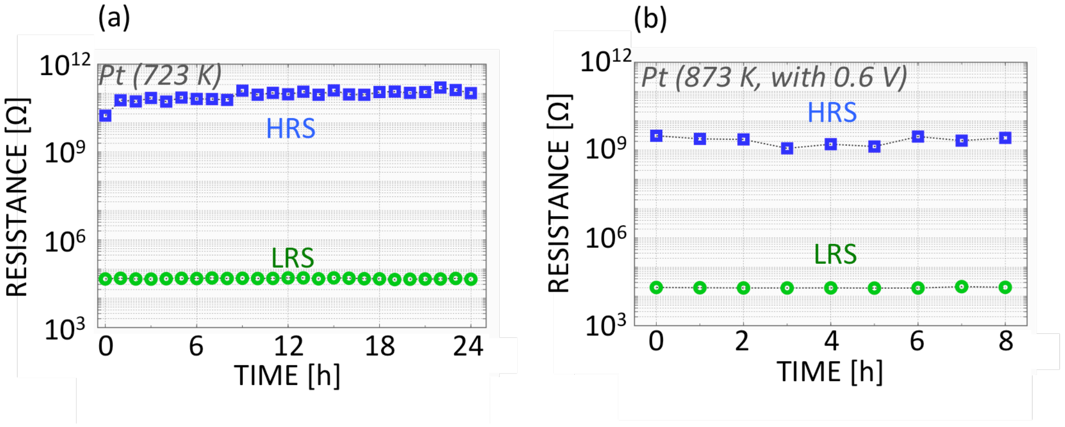

Fig. SI3 Data retention of the two states at (a) 723 K and (b) 873 K. The resistance measurements were recorded at a read voltage of 0.6 V.

The stabilities of the ON and OFF states at high temperatures are shown in Fig. 3(a) and (b). At 723 K and 873 K, retention over longer than 24 hours and longer than 8 hours, respectively, was observed. These high-temperature results indicate stable operation of the NGS memory. For the long-term retention measurements, because of

the high-temperature tolerance of our system, the experimental duration was limited to 8 hours.

4. Material and nanogap shape dependence of switching reproducibility

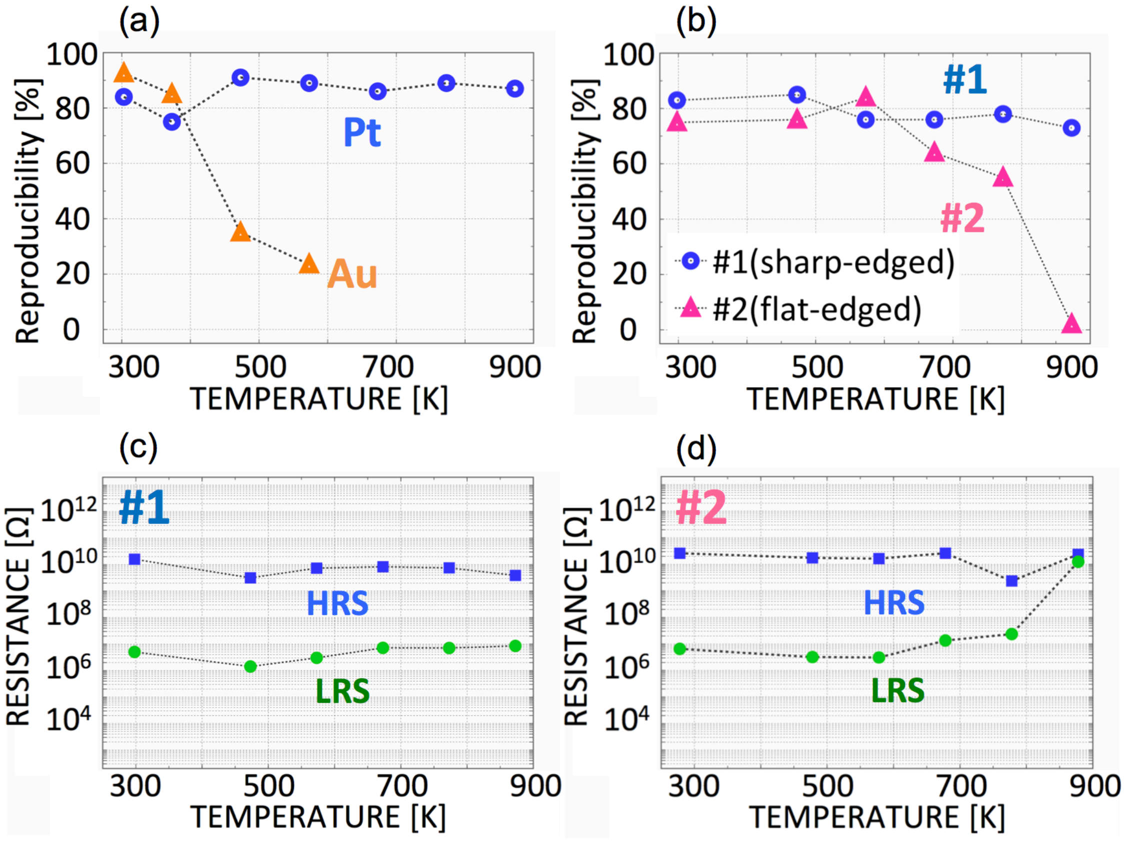

Fig. SI4 (a) Switching reproducibility of Au and sharp-edged Pt (#1 in Fig. 5) nanogaps at various temperatures. (b) Comparison of the sharp-edged (#1 in Fig. 5) and flat-edged (#2 in Fig. 5) Pt nanogaps. (c, d) Temperature dependences of the average resistance values of the LRS and HRS for (c) sharp-edged and (d) flat-edged Pt nanogaps. The average resistances were extracted from 100 cyclic measurements for each resistance.

The temperature dependences of the switching reproducibility were compared for Au and two types of Pt nanogaps. Higher productivity was achieved in Pt than in Au because this higher-melting-temperature material can maintain a rigid contact structure at higher temperatures. In the comparison between the flat-edged and sharp-edged Pt nanogaps, the sharp-edged gap showed better reproducibility at high temperatures because a sharp edge can densify the electric field, enhancing the assembly of the migrating Pt atoms into an electron-emissive needle in the nanogap.

5. Temperature dependence of resistance

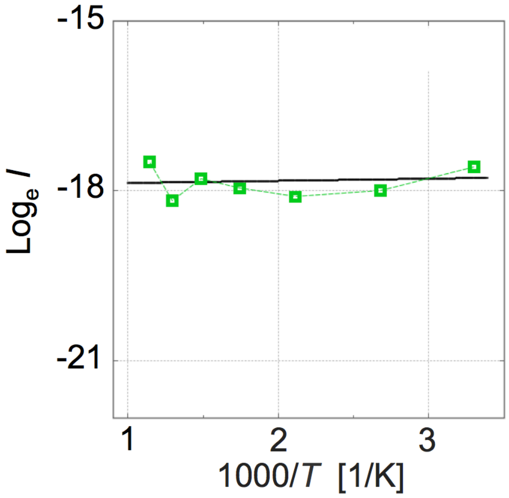

Fig. SI5 Arrhenius plot for the LRS in a polycrystalline Pt nanogap at a bias of +0.6 V from 300 to 873 K.

Figure SI5 shows an Arrhenius plot for the LRS in a polycrystalline Pt nanogap. Although the resistance is extremely small, the plot is nearly flat (Ea ~4 meV), which

suggests that conduction occurs via tunneling and that no metallic contact is formed. These results are consistent with those reported for Au nanogaps in our previous study.[12, SI1] Therefore, we consider that the main LRS conduction mechanism in this study was tunneling and that metallic contacts were not formed.

6. Device-to-device, cycle-to-cycle uniformity

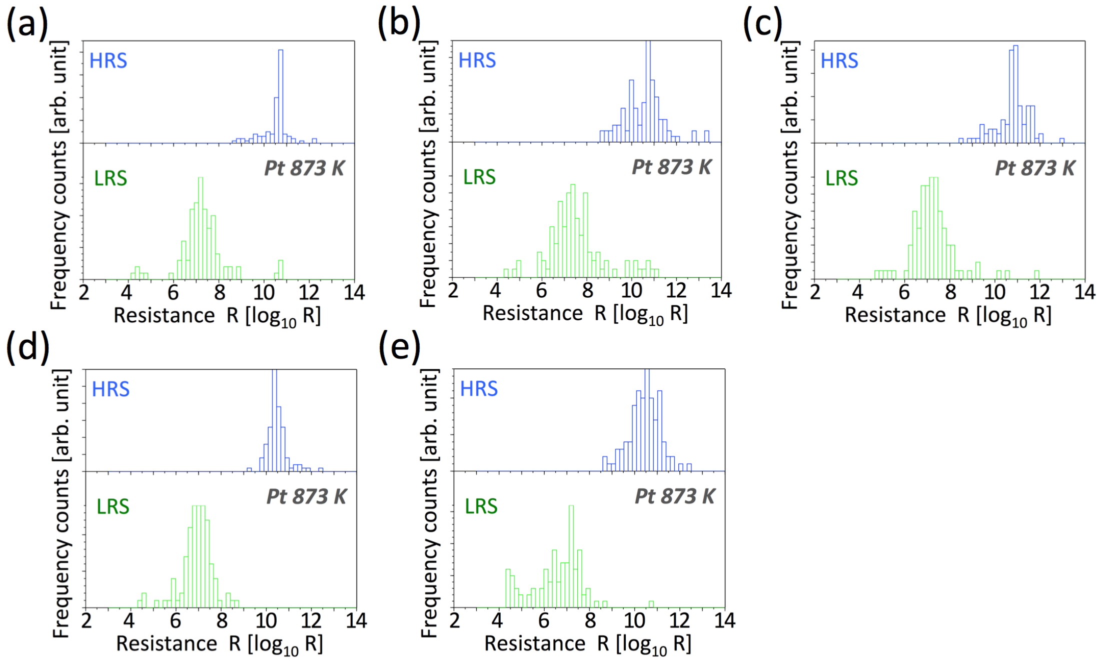

Fig. SI6 Histograms of resistances at 0.6 V. The resistances were measured after switching at 873 K. Each of plots (a) to (e) corresponds to one of five different devices.

Typical resistance histograms at 0.6 V are shown in Fig. SI6. The resistances were measured after switching at 873 K. Both device-to-device and cycle-to-cycle uniformity issues were observed for the Pt nanogaps. These may be attributable to variability in the

nanogap structures. However, the majority of the switching operations were achieved with large ON/OFF resistance ratios. These results indicate good reproducibility of high-temperature operations using polycrystalline Pt nanogaps.

REFERENCES

[Ref. SI1] He, J., Tour, J. M., *et al*, Metal-free silicon–molecule–nanotube testbed and memory device, *Nat. Mat.* **5**, 63 (2006).
